# ImmUniverse Consortium: Multi-omics integrative approach in personalized medicine for immune-mediated inflammatory diseases

**DOI:** 10.3389/fimmu.2022.1002629

**Published:** 2022-11-09

**Authors:** Stefania Vetrano, Gerben Bouma, Robert J. Benschop, Thomas Birngruber, Antonio Costanzo, G. R. A. M. D’Haens, Loredana Frasca, Rainer Hillenbrand, Lars Iversen, Claus Johansen, Arthur Kaser, Hans J. P. M. Koenen, Christa Noehammer, Laurent Peyrin-Biroulet, Jeroen Raes, Leonardo Ricotti, Philip Rosenstiel, Venkata P. Satagopam, Stefan Schreiber, Severine Vermeire, Andreas Wollenberg, Stephan Weidinger, Daniel Ziemek, Silvio Danese, ImmUniverse Consortium, Alessandro Armuzzi

**Affiliations:** ^1^ Department of Biomedical Sciences, Humanitas University, Pieve Emanuele, Italy; ^2^ IBD Unit, Department of Gastroenterology, IRCCS Humanitas Research Hospital, Rozzano, Italy; ^3^ Clinical Pharmacology and Experimental Medicine, GlaxoSmithKline R&D, United Kingdom; ^4^ Immunology and Translation, Eli Lilly and Company, Indianapolis, IN, United States; ^5^ Joanneum Research GmbH, HEALTH - Institute for Biomedicine and Health Sciences, Graz, Austria; ^6^ Dermatology Unit, IRCCS Humanitas Research Hospital, Rozzano, Italy; ^7^ Department of Gastroenterology and Hepatology, Academisch Medisch Centrum Bij De Universiteit Van Amsterdam, Amsterdam, Netherlands; ^8^ Pharmacological Research and Experimental Therapy Unit, Istituto Superiore Di Sanità, Roma, Italy; ^9^ Novartis Pharma AG, Basel, Switzerland; ^10^ Department of Dermatology, Aarhus Universitetshospital, Aarhus, Denmark; ^11^ Aarhus Universitet, Aarhus C, Denmark; ^12^ Division of Gastroenterology and Hepatology, Department of Medicine, University of Cambridge, Addenbrooke's Hospital, Cambridge, United Kingdom; ^13^ Department of Laboratory Medicine, Laboratory of Medical Immunology, Stichting Radboud Universitair Medisch Centrum, Nijmegen, Netherlands; ^14^ Department of Health and Environment, AIT Austrian Institute of Technology GmbH, Vienna, Austria; ^15^ Department of Gastroenterology, Centre Hospitalier Regional Universitaire Nancy, Nancy, France; ^16^ Department of Microbiology and Immunology, Vib Vzw, Gent, Belgium; ^17^ The BioRobotics Institute, Scuola Superiore Sant’Anna, Pisa, Italy; ^18^ Department of Excellence in Robotics & AI, Scuola Superiore Sant’Anna, Pisa, Italy; ^19^ Institute of Clinical Molecular Biology, Christian Albrechts University and University Hospital, Kiel, Germany; ^20^ Luxembourg Centre for Systems Biomedicine, House of Biomedicine II, University of Luxembourg, Belvaux, Luxembourg; ^21^ Department of Internal Medicine I, University Hospital Schleswig-Holstein, Kiel University, Kiel, Germany; ^22^ Department of Gastroenterology and Hepatology, Katholieke Universiteit Leuven, Leuven, Belgium; ^23^ Department of Dermatology, Ludwig-Maximilian-University Munich, Munich, Germany; ^24^ Department of Dermatology, Free University Brussels, University Hospital Brussels, Brussels, Belgium; ^25^ Department of Dermatology and Allergy, University Hospital Schleswig-Holstein, Kiel, Germany; ^26^ Worldwide Research and Development, Pfizer Pharma, Berlin, Germany; ^27^ Department of Gastroenterology and Endoscopy IRCCS Ospedale San Raffaele, Milan, Italy; ^28^ University Vita-Salute San Raffaele, Milan, Italy

**Keywords:** ulcerative colitis, atopic dermatitis, omic analyses, immunomediated diseases, liquid biopsy, dOFM, LIPUS

## Abstract

Immune mediated inflammatory diseases (IMIDs) are a heterogeneous group of debilitating, multifactorial and unrelated conditions featured by a dysregulated immune response leading to destructive chronic inflammation. The immune dysregulation can affect various organ systems: gut (e.g., inflammatory bowel disease), joints (e.g., rheumatoid arthritis), skin (e.g., psoriasis, atopic dermatitis), resulting in significant morbidity, reduced quality of life, increased risk for comorbidities, and premature death. As there are no reliable disease progression and therapy response biomarkers currently available, it is very hard to predict how the disease will develop and which treatments will be effective in a given patient. In addition, a considerable proportion of patients do not respond sufficiently to the treatment. ImmUniverse is a large collaborative consortium of 27 partners funded by the Innovative Medicine Initiative (IMI), which is sponsored by the European Union (Horizon 2020) and in-kind contributions of participating pharmaceutical companies within the European Federation of Pharmaceutical Industries and Associations (EFPIA). ImmUniverse aims to advance our understanding of the molecular mechanisms underlying two immune-mediated diseases, ulcerative colitis (UC) and atopic dermatitis (AD), by pursuing an integrative multi-omics approach. As a consequence of the heterogeneity among IMIDs patients, a comprehensive, evidence-based identification of novel biomarkers is necessary to enable appropriate patient stratification that would account for the inter-individual differences in disease severity, drug efficacy, side effects or prognosis. This would guide clinicians in the management of patients and represent a major step towards personalized medicine. ImmUniverse will combine the existing and novel advanced technologies, including multi-omics, to characterize both the tissue microenvironment and blood. This comprehensive, systems biology-oriented approach will allow for identification and validation of tissue and circulating biomarker signatures as well as mechanistic principles, which will provide information about disease severity and future disease progression. This truly makes the ImmUniverse Consortium an unparalleled approach.

## Introduction

Personalized medicine is the innovative care challenge of 21th century, relying on a large number of cutting-edge research tools, data and technologies and aiming to obtain a better diagnosis and follow-up than the generic “one size-fits-all” model. In 2017, the Food and Drug Administration (FDA) approved a record number of 16 new personalized drugs, kicking off the launch of this new approach to treat many diseases including complex ones, which are caused by a combination of immunological, genetic, environmental, and lifestyle factors, most of which have not yet been identified (https://www.fda.gov/home). Each disease is associated with a large variety of triggering factors, leading to heterogeneity in disease manifestation and progression. However, despite the enthusiasm and the advances made so far, there is still a long way to go to achieve the goals of personalized medicine. Immune-mediated inflammatory diseases (IMIDs) are an example of complex disorders for which a tailored approach is necessary to address the needs of individuals or stratified patient groups (1). IMIDs are a group of disabling, multifactorial unrelated conditions characterized by a dysregulated immune response leading to chronic inflammation and organ damage (2). The immune dysregulation can affect diverse organ systems: gut (e.g., inflammatory bowel disease), joint (e.g., rheumatoid arthritis), skin (e.g., psoriasis, atopic dermatitis) resulting in significant morbidity, reduced quality of life, progressive organ damage, and, in some cases, slight increase in mortality compared to healthy population (1). Though there is evidence emerging that IMIDs share common pathways, we have a poor understanding of the immune factors driving these chronic progressive diseases. The growing incidence of IMIDs in industrial societies supports the hypothesis that economic growth and industrialization are key components of the environmental changes, which combined with a genetic background trigger the IMIDs onset (3). Certainly, socio-economic progress has impacted diet and lifestyle that may have perturbed the complex and delicate network between gut microbiota and host immunity, leading to disturbances of immune system homeostasis such as atopic dermatitis and inflammatory bowel diseases. Indeed, failure to induce tolerance to skin and gut bacteria is thought to result in local and systemic immune responses. Several studies demonstrated that alterations in skin and gut microbiota may have negative consequences on autoimmune diseases (4, 5). Therefore, a better understanding of the complex mechanisms underlying the intricate coordination between host tissues, host immune cells and the resident microbes is crucial for prevention, diagnosis and treatment of IMIDs.

Large unmet clinical treatment needs remain for many IMIDs. An optimal therapeutic approach for these diseases should gain rapid control of inflammation and prevent secondary consequences such as tissue damage or comorbidities, thus improving patient’s quality of life and, if possible, achieving long-term disease remission. Although several advanced therapies, mainly monoclonal antibodies and small molecules, do provide clinical benefits to patients (6, 7), long term disease control is still poorly met. Improving our knowledge of the underlying mechanisms of the immunopathology and, in particular, identification of biomarkers that would allow prediction of disease progression, clinical/therapy outcome and proper patient stratification, are therefore urgently needed.

Ulcerative colitis (UC) and atopic dermatitis (AD) are examples of IMIDs that are debilitating, burdensome, and chronic conditions characterized by dysregulated immune responses leading to destructive chronic inflammation.

UC is a chronic, idiopathic form of immune-mediated intestinal disease that, unlike Crohn’s disease, affects specifically the colon. UC most commonly affects young adults (usually at the age of 20–40 years) and results in disability and lower quality of life (8, 9). It is characterized by relapsing and remitting mucosal inflammation, starting in the rectum and extending to proximal segments of the colon. UC is associated with damage to the mucosal barrier, allowing the luminal microflora to trigger a sustained and uninhibited inflammatory response. Among the inflammatory cells, T helper (Th) cells perpetuate enterocyte apoptosis and inhibit mucosal reparation and healing. IL-13, produced by NK T cells, also contributes to epithelial injury (10, 11). Additionally, innate lymphoid cells, homeostatic at steady state, contribute to the inflammatory cytokine production, thereby perpetuating inflammation. Mucosal injury and damage are also associated with dysbiosis, which possibly further contributes to the inflammatory cascade. Our increasing understanding of the mucosal immune system has led to an expanding array of therapeutic targets. To date, the main therapeutic targets are induction and maintenance of clinical and endoscopic remission (defined as absence of symptoms, and restoration of normal colonic mucosa, respectively) (12). Aminosalicylates are the main choice of treatment for mild to moderate UC, systemic corticosteroids can be used to treat moderate-to-severe flares, whereas small molecules and monoclonal antibodies are used in moderate to severe disease. Colectomy is needed in up to 15% of patients with UC. The annual direct and indirect costs related to UC are estimated to be as high as €12.5–29.1 billion in Europe and US$8.1–14.9 billion in the USA (8). However, the number of drugs modulating different disease pathways is expected to expand in the near future. In spite of the current availability of about 30 different therapeutic molecules belonging to almost 10 different drug classes (13), their impact on the long-term maintenance of deep UC remission (defined as the absence of symptoms, normalization of inflammatory biomarkers and tissue healing), and, in particular, the ability to prevent long-term complications and irreversible damage, remains to be fully understood.

AD is the most common chronic IMID skin disorder with an annual prevalence of around 5% in adults (14) and 12-15% in children and adolescents (15). Up to 50% of adult patients suffer from moderate to severe disease (14, 16). Its impact on overall health and quality of life is comparable to other serious chronic disorders such as diabetes (17), with high medical costs comparable to those of e.g. asthma (18–20), and a considerable impairment of quality of life (16, 21). The burden of AD is further increased by a strongly increased risk for several allergic, inflammatory and psychiatric comorbidities (22–24). The pathophysiology is complex and involves a strong genetic predisposition, epidermal dysfunction, and T-cell driven inflammation, with increased production of inflammatory cytokines, in particular type 2 cytokines such as IL‐4, IL‐13, and IL‐31, but also activation of other immune axes such as Th17 and Th1 (25, 26). However, AD is an extremely heterogeneous disease in terms of clinical features and disease trajectories, and, as a consequence of its traditional definition, multiple disease subtypes and endotypes are likely clustered together into a single phenotype. As a result, the mechanisms reported for AD at large are probably not equally important/pronounced in all patients (27). After many years of little advancements, there is now an expanding therapeutic pipeline, which includes biologics and small molecules, available to the considerable proportion of patients whose disease can not sufficiently be controlled with topical treatment only (6).

## Biological systems: A new approach for diagnosis and therapy

The multifactorial origin and aberrant immune response represent major obstacles for the development of effective therapies for both UC and AD. Indeed, current treatments are not always effective and many patients lose response to drugs over time, most likely due to compensatory mechanisms ultimately leading to drug resistance. Variability in disease modulating response of drugs is most likely driven by the changing state and response of the immune system over time.

Therefore, an appropriate molecular and cellular characterization, or signature, that accounts for the inter-individual differences in severity, drug efficacy, side effects or prognosis would help to guide clinicians in the management of patients and represent a major step towards tailored medicine for these diseases. To achieve this ambitious goal, an advanced methodological approach is needed that would allow molecular and cellular characteristics found in blood and target disease tissue to be put in context with clinical outcome prediction. Indeed, to understand complex biological systems, it is not sufficient to characterize the individual molecular components in the system. It is essential to understand their molecular interactions, which requires integrated analysis of so-called big data from advanced omics technologies (28), efficiently supporting the investigation into how disrupted biological processes cause disease at a molecular level (29–31). The acknowledged pathogenic components, i.e., environment, genes, gut microbiota and the immune system must be viewed as “omes”, a Greek word indicating the totality of something. Each ome is remarkably multifactorial, with thousand to million components, and all of them interact with and impact on the function of each other (31).

Both UC and AD represent two disease areas where routine biosampling of blood and target tissues is more feasible than in other conditions, offering more opportunities for better understanding of their immunopathology and identification of potential new biomarkers. However, the lack of adequate parallel characterization of the circulating immunological signatures and tissue microenvironment limits the use of blood sampling as a liquid biopsy.

## International efforts to overcome the gap of IMIDs

Multiple research groups and consortia throughout the world are generating datasets combining clinical data with either genome data, transcriptome data, or gut microbiome data. By studying these datasets, researchers can address important individual questions. A systematic taxonomy for patients with autoimmune diseases at different omics levels has been supported by the innovative medicine initiative project PRECISESADS (32). Analyzing whole blood samples by using transcriptomic, genomic, epigenomic, cytokine expression and flow cytometry approaches, and combining them with clinical parameters, new insights into a better stratification of patients with systemic sclerosis, syndrome Sjögren’s, systemic lupus erythematosus, and rheumatoid arthritis have been achieved (33–37). As a results, our understanding of the pathophysiologic mechanisms underlying these diseases has improved and potential biomarkers helping to design personalized treatment have been identified.

In addition, results from (38) IMID multi-omics projects already exist in the longitudinal integrative Human Microbiome Project the PRISM-cohort (39), and the RISK-cohort (40) in the United States.

Previous efforts from the RISK cohort, the PRISM cohort, as well as from consortia in which these cohorts participate, have already enabled the first steps towards precision medicine in inflammatory bowel disease (IBD), including UC. For example, microbial DNA profiles and RNA-sequencing profiles from the intestinal biopsies of the RISK cohort have uncovered RNA-microbe interactions and shown that biopsies taken from the distal colon can predict the IBD disease location higher up in the intestine (40, 41). In addition, stool samples from the PRISM cohort have also been used to discover microbial profiles that can predict the efficacy of vedolizumab, a biological drug regulating T-cell homing to the gut (39). Another important project named the 1000IBD project has been initiated in Europe to prospectively follow more than 1000 IBD patients from the Northern provinces of the Netherlands. For these patients, a uniquely large number of phenotypes has been collected and multi-omics profiles have been generated (42). To date, 1215 participants have been enrolled in the project and the enrolment is still on-going. The aim of the 1000IBD project is to discover molecular subtypes and biomarker profiles that would capture the clinical heterogeneity of IBD and to prioritize new targets for early-stage drug discovery or other interventional strategies.

Despite increased research efforts in recent years, most omics-level data in AD concern only genomics and transcriptomics. There are some novel proteomic (in serum) and lipidomic (in sweat samples) studies showing great promise toward identifying AD endotypes (29). Recently, BIOMAP, a multidisciplinary international project on AD and psoriasis has been initiated, with the aim to refine clinical phenotypes and to define relevant and patient-centered outcomes, identify genetic risk factors as well as influential life events and environmental factors, define endotypes and associated molecular signatures, and generate predictive models and biomarkers of relevant disease outcomes (27). The strategies used in Biomap will inform and inspire ImmUniverse (43).

## ImmUniverse solution: Multi integrative approach

**Figure 1:**
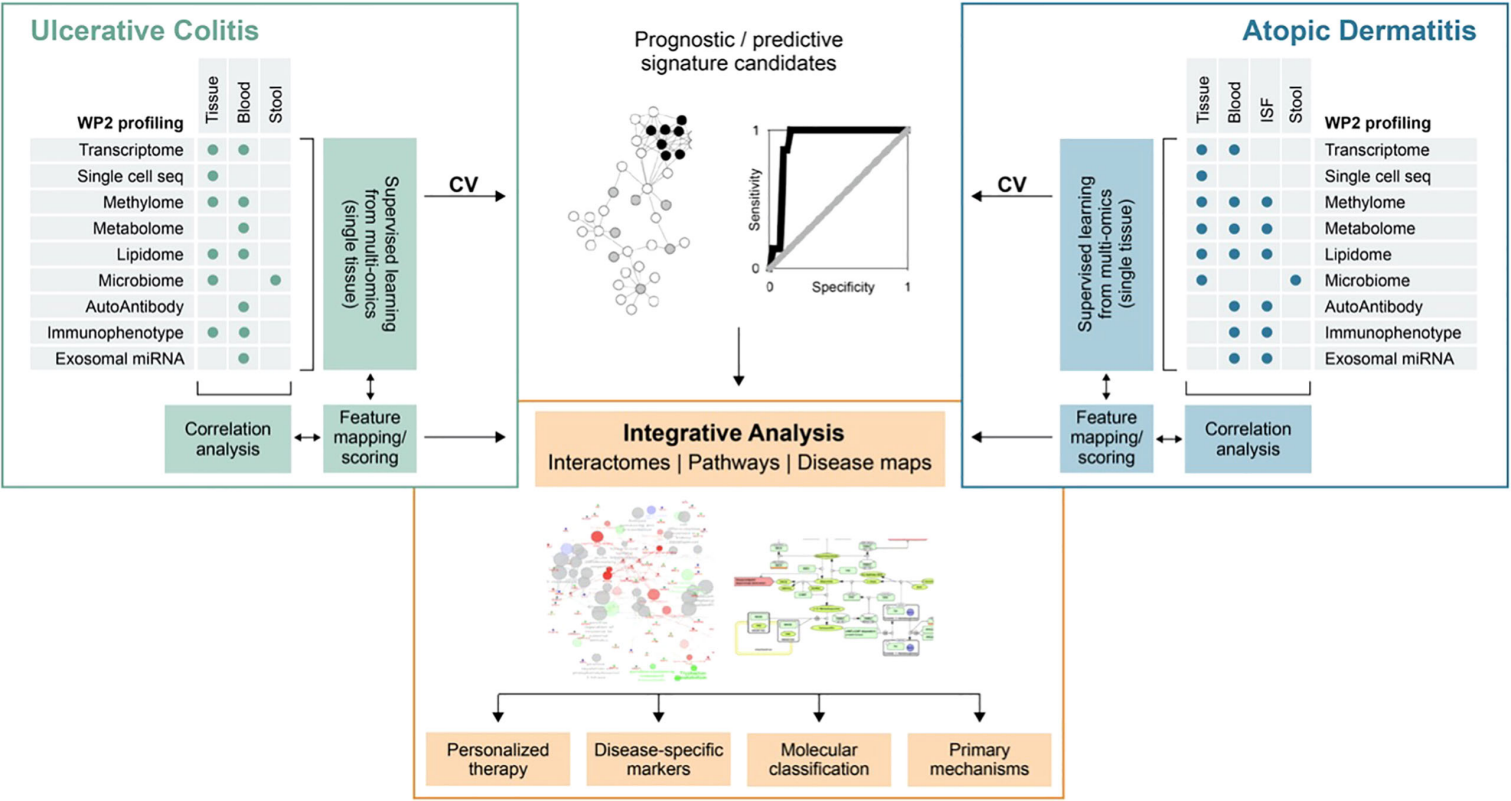
Schematic diagram of ImmUniverse data integration strategy. Ulcerative colitis and atopic dermatitis will be analyzed separately according to the same principle: First, supervised multi-omics analysis will be performed to identify candidate prognostic and predictive signatures from multi-omics analysis of single tissue data and evaluate their performance in cross-validation (CV) or through publicly available data (as available). Signatures will be then correlated between sample matrices to identify ‘proxy’ – signatures in matrices suitable for clinical exploitation. Features from prediction and correlation tasks will be pooled, mapped to pathways and ontologies and prioritized on the basis of their importance for prediction, across-tissue correlation and pathway annotation in a collaborative effort between project partners (clinicians, biologists and bioinformaticians). Comparison between UC and AD will be done subsequently on the basis of shared and specific pathways and ontologies. From these, interaction networks and disease maps will be generated to describe the overlapping and unique processes in the relevant tissue types. ISF, interstitial fluid.

ImmUniverse is a large collaborative consortium that received funding from the Innovative Medicines Initiative (IMI), which is sponsored by the European Union (Horizon 2020), and in-kind contributions from participating pharmaceutical companies within the European Federation of Pharmaceutical Industries and Associations (EFPIA). ImmUniverse is a partnership between academic experts in UC and AD, technical expert providers, patient organizations and large pharmaceutical companies (Annex 1), aiming to advance our understanding of the molecular and cellular mechanisms underlying both UC and AD by implementing a multi-omics approach in both blood and disease tissue. The novelty of this research project is to combine state-of-the-art omics profiling and informatics expertise to identify signatures of tissue-derived and circulating biomarkers and elucidate mechanistic principles that are informative for disease severity and future disease progression of these 2 different IMIDs. To this end, the study will follow a registry-like design, with physician choice of targeted therapies and will recruit >500 UC and AD adult patients (male and female patients ≥ 18 years of age) with complete data including a clinical follow up over at least 12 months. Multi-omics data from different molecular profiling of tissue microenvironment and accessible matrices, such as blood and stool, using multiple technologies (single cell sequencing, multi-omics approaches) will be integrated with clinical data. Signatures identified in blood or other matrices will be correlated with signatures in the tissue. The ultimate goal is therefore two-fold: (A) Integration of different omics layers on single tissue data to derive predictive and prognostic multi-omics signatures and (B) identification of signatures correlating between tissue and blood. Multi-omics predictive models will undergo thorough crossvalidation to derive robust estimates for classification performance, which will be benchmarked against comparable single-omics performances and scrutinized by ImmUniverse’s clinical team for their clinical utility. Reported will be not only the performance estimates, but also the predictive features (signatures) including a measure of their respective importance for classification. Tissue-derived signatures will be correlated with data derived from accessible matrices (blood and stool), using variations of canonical correlation analysis capable of dealing with the dimensionality of the data. Again, such circulating features shall be reported for the subsequent task of enriching the detected features with data from open sources. Tissue-specific molecular interaction networks and pathway maps will be identified and visualized for both diseases and compared for the tissue-specific determination of shared and disease-specific processes and pathways. Hormonal and genetic factors contribute significantly to sex differences in immune function and disease pathogenesis. Along these lines ImmUniverse will systematically take into account biological sex differences as well as cultural/behavioral influences from phenotype/outcome definitions to tissue-based molecular analyses through to data models. Therefore, we will consider the potential confounding influence of gender at all stages of analysis and incorporate gender as a co-variate. Based on these features and enriched with data from the literature, a disease map will be developed in close collaboration with clinical partners and domain experts, which will describe the mechanisms of the pathophysiology behind the investigated diseases, highlighting their overlapping and specific areas in the context of specific tissues. This approach will not only generate translatable signatures for disease prognosis and therapy response prediction in UC and AD, but it will also develop a first mechanistic hypothesis of the underlaying pathophysiologies (both shared and disease-specific). This integrated analysis approach is depicted in **Figure 1**. Aggregated results will be shared with public *via* scientific publications and patients/patient organizations. Patients have right to request their data and we will securely share the corresponding data with requested study participant. Researchers will be able to search the aggregated metadata indexed in the data catalogue and submit a request to Data Access Committee (DAC) to access necessary datasets. Once the DAC approves the request, the requested data will be securely shared with the researcher.

The inclusive mechanistic models and algorithms for the integration and analysis of tissue, clinical, and omics data will be used as variables in the identification of classifiers and predictors of disease progression. Indeed, from the aggregation of multidimensional omics data, ImmUniverse will select biomarker candidates based on the following criteria: (A) ‘translatability’, i.e. can a test based on a marker (signature) be translated into the clinical routine (tissue accessibility, technical feasibility), (B) clinical utility based on the clinical need and the performance estimates of a candidate marker (signature) and (C) biological relevance based on the disease models & maps and interactivity networks for key regulatory genes controlling the downstream molecular alterations observed in the omics data using a causal reasoning analysis.

While the extensive retrospective availability of clinical data and biomaterial will aid in a hypothesis-building approach, non-interventional prospective longitudinal therapy cohorts of patients with UC and AD will be another essential key for this project to ensure that identified signatures are clinically relevant.

Only a large trans-disciplinary, collaborative network can succeed in the discovery of individual disease and cross-disease biomarkers of disease progression. Therapy monitoring and integration of the multiple layers of data will help to gain a better understanding of the underlying complex and often heterogeneous biology of IMIDs.

**Figure 2:**
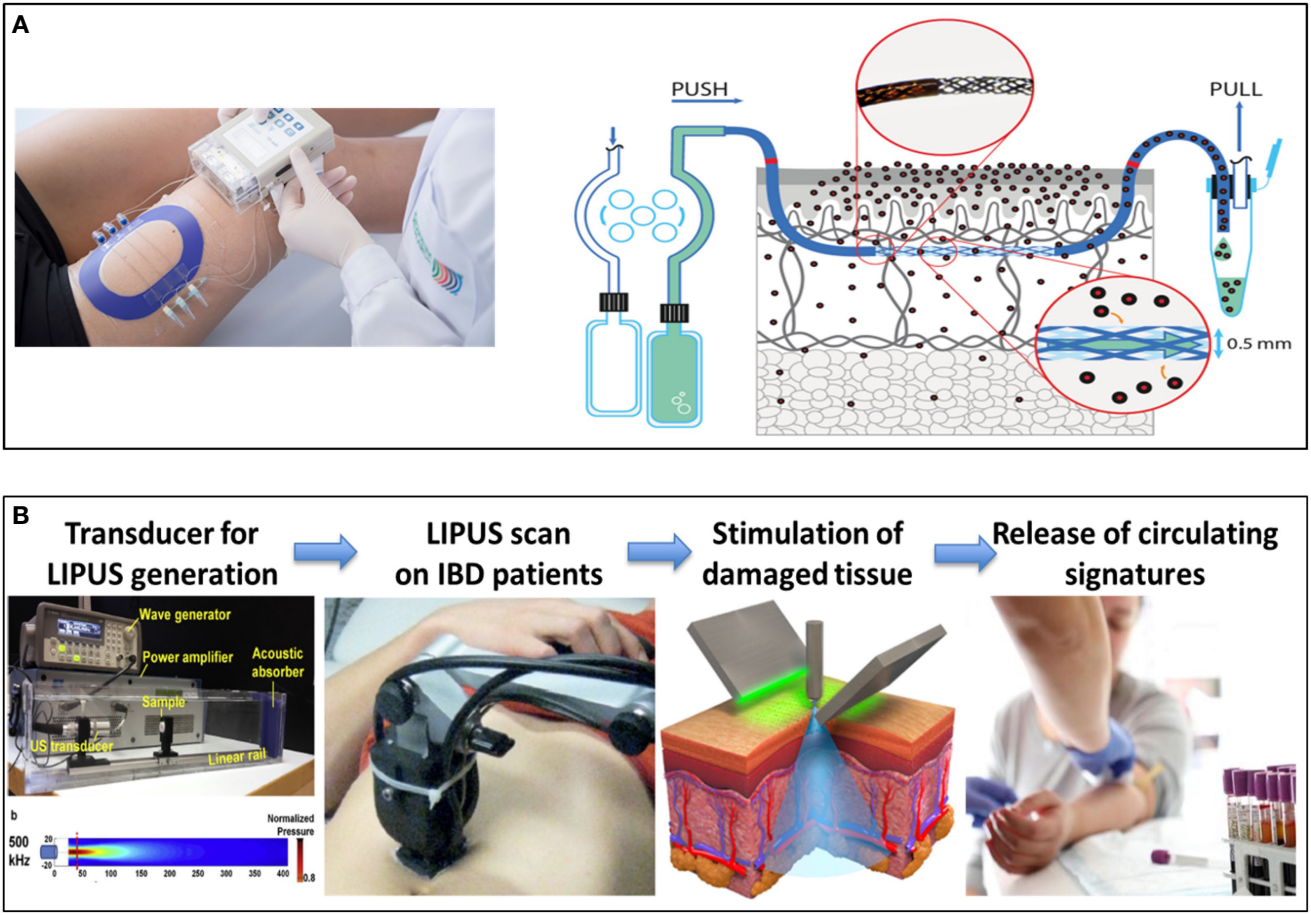
Development of new Disruptive liquid biopsy technologies. Schematic overview of dermal open-flow microperfusion (dOFM) **(A)** and LIPUS-enabled gut liquid biopsy **(B)** as non-invasive approach for the identification of new signatures in the skin and in the gut. dOFM is a sampling method for clinical and preclinical drug development studies and biomarker research. OFM is designed for continuous sampling of analytes from the interstitial fluid (ISF) of various tissues. It provides direct access to the ISF by insertion of a small, minimally invasive, membrane-free probe with macroscopic openings. Thus, the entire biochemical information of the ISF becomes accessible regardless of the analyte’s molecular size, protein-binding property or lipophilicity. Therefore dOFM allows a new level of insight into physiological and pathophysiological processes directly in the skin. LIPUS is a versatile and non-invasive tool, recently explored for the treatment of a variety of medical conditions. It consists of ultrasound waves that bring mechanical energy with a characteristic frequency higher than the limit of human hearing (20 kHz), a typical intensity smaller than 3 W/cm2 and a pulsed mode. By depositing mechanical or thermal energy in the target tissues, this particular ultrasound regime can trigger a plethora of different beneficial bioeffects, by exploiting cell mechanotransduction mechanisms, without harmful effects. The therapeutic effects of LIPUS are mostly associated to low mechanical events such as stable cavitation, acoustic streaming and acoustic radiation force, while thermal effects are often negligible (typical temperature increases are smaller than 1°C). LIPUS is currently approved by FDA for the treatment of fracture healing (36), and other therapeutic effects have been repeatedly demonstrated *in vitro* and *in vivo* on different types of tissues (37) although the optimization of the procedures and the knowledge of the underlying mechanisms still need to be elucidated. The studies available on LIPUS are almost always based on rather uncontrolled and often unknown ultrasound stimulation protocols, only few studies correlate a precise LIPUS dose with the bioeffects achieved (38, 39). Therefore, proper instruments and a correct methodology are crucial to obtain reliable ultrasound measurements, exposure estimations and bioeffect optimization *in vitro*, to be then effectively translated *in vivo*. ImmUniverse will set up a LIPUS device with peculiar characteristics to induce bioeffects on the gut allowing the release of mediators in the blood.

The overall concept of identifying integrated comprehensive signatures of disease-affected tissue microenvironments and matched blood biosamples over time will be supported by disruptive non-invasive liquid-biopsy methodologies to ultimately reduce the reliance on invasive biopsy methods. Two non-invasive technologies, dermal open-flow microperfusion (dOFM) in the skin (**Figure 2A**) and low-intensity pulsed ultrasound stimulation (LIPUS) in the gut (**Figure 2B**), will be set up as disruptive liquid biopsy technologies. These will be applied in a translational setting to establish specific disease signatures in blood and explore such novel technologies to overcome currently invasive biopsy methods and improve the patient experience. dOFM has the potential to provide a high resolution signature of the interstitial fluid as the intermediary component between tissue and blood (44–47), to allow correlation between tissue and blood and aid in the identification of robust circulating signatures in AD. LIPUS has the capacity to induce release of tissue-specific cellular and molecular components into the blood, allowing induction of circulating signatures and thereby replacing traditional intestinal biopsies. Currently, biopsy and endoscopic assessment of mucosal healing are the gold standard for diagnosis of inflammatory bowel disease and the monitoring of disease progression. Although generally well tolerated, both endoscopy and biopsies are invasive procedures and, consequently, their use and frequency is limited. Furthermore, they are limited in their ability to truly capture disease dynamics throughout the colon or capture patients response or sensitivity to the treatment due to the limited sampling that is tolerated. Moreover, traditional biopsies are limited by the quality and amount of the tissue that can be sampled. Similarly, in dermatology the frequency of biopsy sampling is a limiting factor due to their invasive nature. Therefore, there is a high clinical need for robust signatures of the disease tissue microenvironment from blood and/or non-invasive detection methods that allow better monitoring of the real-time dynamics of IMIDs. The liquid biopsy represents an alternative and attractive non-invasive procedure.

ImmUniverse aims to significantly improve IMID management and provide benefit to patient’s life and well-being. Following a unique and unparalleled approach, ImmUniverse will address the limitations of previous investigational approaches by investigating the complex interactions between circulating immune cells and tissue microenvironment in an integrated manner. Due to the parallel study of two different IMIDs, the project will enable identification of both disease-specific as well as cross-disease signatures and the underlying pathological pathways. ImmUniverse will provide a scalable, clinic-ready production infrastructure for delineating the tissue microenvironment and accessible matrices, such as blood and stool, using multiple technologies (single-cell sequencing, multi-omics approaches) on several molecular omics layers. The ImmUniverse project will bring IMID clinical management to a new level through novel, validated and clinic-ready circulating biomarker assays which are expected (a) to improve diagnosis, (b) to inform early in the clinical course on disease severity and progression and (c) to enable better treatment response/remission monitoring. Moreover, implementing disruptive non-invasive liquid-biopsy methodologies will provide significant advances to patients by reducing the reliance on invasive biopsies. The complex and ambitious nature of the project requires the unique collaborative nature of the ImmUniverse consortium to bring together deep expertise and experience in disease understanding, technology, clinical trials and patient management from academia, technology experts, industry partners and patients themselves.

## Conclusions and outlook

IMIDs are heterogeneous diseases for which, despite advancement of targeted therapies, large unmet need remains. Differentiation of targeted treatments is limited by the lack of clear molecular, cellular or clinical characteristics that could be used to identify patient subpopulations and allow more tailored treatment. In general, the rate of primary non-response is high (25-40%) and more than 50% of the initial responders fail to reach long-term remission. With more targeted therapies becoming available, new approaches are needed to bring IMIDs, in particular UC and AD, to a 21th century level of investigation and understanding that would allow us to move towards tailor-made treatments for patients, maximizing the likelihood of therapeutic efficacy and minimizing the risk of side effects. In view of the overwhelming complexity of molecular and cellular components driving pathology, there is a need for improved, comprehensive and systematic analyses of the individual components through their biological integration at a molecular and cellular level. ImmUniverse will fill this gap and aims to not only identify disease-specific, but also cross-disease signatures of underlying pathological pathways. By this, ImmUniverse will provide a scalable production infrastructure for delineating the tissue microenvironment and accessible matrices, such as blood and stool, using multiple technologies on several molecular Omics layers. In addition, the validation of the use of non-invasive liquid-biopsy methodologies for molecular characterization of skin and gut will pave the way to novel approaches for monitoring treatment response and disease progression.

## Data availability statement

The original contributions presented in the study are included in the article/**Supplementary Material**. Further inquiries can be directed to the corresponding authors.

## Author contributions

SeV, GB, and SD conceived, guided, and wrote the article, and all members of the ImmUniverse consortium discussed and conceived content of this article and contributed to its writing. All authors contributed to the article and approved the submitted version.

## Funding

This ImmUniverse (www.immuniverse.eu) has received funding from the Innovative Medicines Initiative 2 Joint Undertaking (JU) under grant agreement No. 853995. The JU receives support from the European Union’s Horizon 2020 research and innovation program and EFPIA. The content provided in this publication reflects only the author’s view and neither the IMI JU nor the European Commission are responsible for any use that may be made of the information it contains.

## Acknowledgments

The authors thank European Research and Project Office GmbHfor her help with graphic design, and European Federation Of Asthma & Allergy Associations Ideell Forening (EFA), European Federation Of Crohn’S And Ulcerative Colitis Associations (EFCCA), and Forum Des Patients Europeens (EPF) for revising the manuscript.

## Conflict of interest

GB was employed full time by the company GSK at the time of writing; RJB was employed full time by the company Eli Lilly and Company; DZ was employed full time by the company Pfizer Limited; RH was employed full time by the company Novartis Pharma AG.

The remaining authors declare that the research was conducted in the absence of any commercial or financial relationships that could be construed as a potential conflict of interest.

## Publisher’s note

All claims expressed in this article are solely those of the authors and do not necessarily represent those of their affiliated organizations, or those of the publisher, the editors and the reviewers. Any product that may be evaluated in this article, or claim that may be made by its manufacturer, is not guaranteed or endorsed by the publisher.
