# Supplementary material for: ImmUniverse Consortium: Multi-omics integrative approach in personalized medicine for immune-mediated inflammatory diseases
Source: Front Immunol. 2022 Nov 9;13:1002629. doi: 10.3389/fimmu.2022.1002629 (PMC9682955; doi:10.3389/fimmu.2022.1002629)
Supplement: Supplementary file 1 [file DataSheet_1.docx]

**SUPPLEMENTAL INFORMATION**

**List of ImmUniverse consortium members and affiliations**

Alessandro Armuzzi^1^, Alessia Butera^6^, Andrea Cafarelli^15,16^, Lucia Ciglar^12^, Ellen van den Bogaard^11^, Elke MGJ de Jong^11^, Ernst Dow^3^, Marjolijn Duijvenstein^11^, Ahmed Essaghir^2^, Rebecca Favaro^1^, Gionata Fiorino^24^, Soumyabrata Ghosh^18^, Wei Gu^18^, Robert Häsler^22^, Xuehui He^11^, Roberto Lande^6^, Judith Logmans^11^, Christoph Magnes^4^, Karl Nocka^23^, Loulou Peisl^21^, Samuel Pineda Chavez^1^, Angela Sorriento^15-16^, Klemens Vierlinger^12^, Laurien Waaijer^11^, Xinhui Wang^18^, Nicolas Wisniacki^2^, Mary Zuniga^3^.

(1)Department of Biomedical Sciences, Humanitas University, Pieve Emanuele (Milan), Italy and IBD Unit, Department of Gastroenterology, IRCCS Humanitas Research Hospital, Rozzano, Milan, Italy

(2)Clinical Pharmacology and Experimental Medicine, GlaxoSmithKline R&D, Gunnels Wood Road, SG1 2NY, Stevenage, UK

(3) Eli Lilly and Company, Indianapolis, IN, USA

(4)Joanneum Research GmbH, HEALTH - Institute for Biomedicine and Health Sciences, Neue Stiftingtalstrasse 2, 8010 Graz, Austria

(5)Department of Biomedical Sciences, Humanitas University, Pieve Emanuele (Milan), Italy and

IBD Unit, Dermatology Unit, IRCCS Humanitas Research Hospital, Rozzano, Milan, Italy

(6)Department of Gastroenterology and Hepatology. Academisch Medisch Centrum Bij De Universiteit Van Amsterdam, Amsterdam, Netherlands

(7)Pharmacological Research and Experimental Therapy Unit.Istituto Superiore Di Sanità, Roma, Italy

(8)Novartis Pharma AG, Basel, Switzerland

(9)Department of Dermatology. Aarhus Universitetshospital, Aarhus, Denmark

(10)Aarhus Universitet, Aarhus C, Denmark

(11)Division of Gastroenterology and Hepatology, Department of Medicine, University of Cambridge, Addenbrooke's Hospital, Cambridge, UK

(12)Department of Laboratory Medicine, Laboratory of Medical Immunology. Stichting Radboud Universitair Medisch Centrum, Nijmegen, Netherlands

(13)AIT Austrian Institute of Technology GmbH, Vienna, Austria

(14)Department of Gastroenterology.Centre Hospitalier Regional Universitaire Nancy, Nancy Cedex, France Christian-Albrechts

(15)Department of Microbiology and Immunology. Vib Vzw, Zwijnaarde - Gent, Belgium

(16)The BioRobotics Institute, Scuola Superiore Sant’Anna, Piazza Martiri della Libertà 33, 56127 Pisa, Italy

(17)Department of Excellence in Robotics & AI, Scuola Superiore Sant’Anna, Piazza Martiri della Libertà 33, 56127 Pisa, Italy

(18)Institute of Clinical Molecular Biology, Christian Albrechts University and University Hospital, Kiel, Germany.

(19)Luxembourg Centre for Systems Biomedicine, University of Luxembourg, Campus Belval, House of Biomedicine II, 6 avenue du Swing, L-4367 Belvaux, Luxembourg

(20)Department of Internal Medicine I, University Hospital Schleswig-Holstein, Kiel University, Kiel, Germany.

(21)Department of Gastroenterology and Hepatology.Katholieke Universiteit Leuven, Leuven, Belgium

(22)Department of Dermatology, Ludwig-Maximilian-University Munich, Munich, Germany and Department of Dermatology, Free University Brussels, University Hospital Brussels, Brussels, Belgium

(23)Department of Dermatology and Allergy, University Hospital Schleswig-Holstein, Kiel, Germany

(24)Pfizer Pharma, Worldwide Research and Development, Linkstraße 10, 10785, Berlin, Germany

(25)Department of Gastroenterology and Endoscopy IRCCS Ospedale San Raffaele

(26) University Vita-Salute San Raffaele, Milan, Italy
